# Beneficial effects of combined administration of Clopidogrel and Aspirin on the levels of proinflammatory cytokines, cardiac function, and prognosis in ST-segment elevation myocardial infarction: A comparative study

**DOI:** 10.1097/MD.0000000000013010

**Published:** 2018-11-09

**Authors:** Hai-Rong Yu, Yue-Yue Wei, Jian-Guo Ma, Xiao-Yong Geng

**Affiliations:** a Department of Functional Experiment Center, Chengde Medical Collage, Chengde; b Department of Urology, The Third Hospital of Hebei Medical University, Shijiazhuang; c Department of Cardiology, The Third Hospital of Hebei Medical University, Shijiazhuang, P. R. China.

**Keywords:** Aspirin, cardiac function, Clopidogrel, inflammatory factors, prognosis, ST-segment elevation myocardial infarction

## Abstract

**Objective:**

Both Aspirin and Clopidogrel are considered as effective drugs in decreasing ischemic events, which potentially contribute to a promising application regarding the cardiovascular events. In the present study, we evaluated the efficacy of the combination of both Clopidogrel and Aspirin to determine the influence among inflammatory factors, cardiac function, and treatment outcome of patients suffering from ST-segment elevation myocardial infarction (STEMI) in the Hebei province of China.

**Methods:**

To compare the efficacy of this combination therapy with a single Aspirin treatment, we experimented in 68 patients with the administration of both Clopidogrel and Aspirin as well as another 68 patients administered only with Aspirin. An enzyme-linked immunosorbent assay was used to measure the expression of inflammatory factors, thereby evaluating the effect on inflammation. In addition, a series of indexes related to cardiac function and renal function were monitored by use of a color Doppler ultrasound and an automatic biochemical analyzer, respectively. Myocardial injury-related indicators were detected. A multivariate logistic regression analysis was performed so we could identify potential risk factors. In addition, both postoperative hemorrhages and cardiac events were observed to evaluate the treatment outcome of patients with STEMI.

**Results:**

Initially, the treatment outcome revealed a better efficacy in patients treated with the combination of both Clopidogrel and Aspirin, with the patients also showing more obviously alleviated myocardial injury, better cardiac and renal functions with lower serum levels of inflammatory factors. The lower incidence of postinfarction angina, recurrent myocardial infarction, stroke, and death also provide evidence that patients showed a better outcome after treatment with both Clopidogrel and Aspirin.

**Conclusion:**

Taken together, the combination therapy of Clopidogrel and Aspirin provided a better improvement on both the cardiac function and outcome of STEMI patients in the Hebei province of China, with reduced inflammation as well.

## 1 Introduction

Myocardial infarction (MI), commonly known as a heart attack, is an irreversible process of the heart muscle necrosis secondary to prolonged ischemia, making it the key medical issue involved with high morbidity and mortality.^[1]^ Among the spectrum of acute coronary syndromes, the most serious result is ST-segment elevation myocardial infarction (STEMI).^[2]^ STEMI is a kind of emergency medical condition and mortality in patients with this disease remains high.^[3]^ The risk factors for STEMI are composed of plaque disruption, platelet aggregation, and intracoronary artery thrombus formation.^[4]^ Inflammatory-related factors including oxidative stress are contributors to the development of cardiovascular diseases, including STEMI.^[5]^ Nitrotyrosine, as an oxidative stress marker,^[6]^ is a modified amino acid, produced through free-radical (O_2_^−^) interaction with nitric oxide,^[7]^ and it is associated with the presence of coronary artery disease.^[8]^ Survival of STEMI patient is up to various factors (prevention of severe arrhythmias and heart failure), and the most important one is spending time to restore the brisk antegrade coronary flow, so as to achieve the desired sustained patency of the infarct-related coronary artery.^[9]^ The general treatment for STEMI patients includes primary percutaneous coronary intervention (PCI) and pharmacologic reperfusion therapy, and PCI decreases infarct size, reinfarction, recurrent ischemia, stroke, and does well to improve survival in comparison to that of the pharmacologic reperfusion therapy.^[10,11]^ After PCI, the reestablishment of normal coronary blood flow becomes one of the most crucial therapeutic challenges in managing STEMI because those who undergo PCI usually run the greatest risk of developing further bleeding complications.^[12,13]^ As a result, we need to uncover more efficient and affordable ways for the treatment of STEMI.

Aspirin is a drug used regularly for the long-term prevention of primary and secondary cardiovascular disease, whereas larger dosages are related to the elevated incidences of bleeding.^[14]^ Therapy involving the use of low-dose and long term aspirin confirmed of reducing the risk of serious vascular events annually (nonfatal myocardial infarction, nonfatal stroke, or vascular death) within approximately a quarter of patients who have already had occlusive vascular disease.^[15]^ Clopidogrel belongs to thienopyridine derivatives, which are antiplatelet agents inhibiting the platelet aggregation caused by adenosine diphosphate, thus decreasing ischemic events.^[16]^ Dogan et al have proved that Aspirin combined with Clopidogrel enhances the effects in both the platelet activation and aggregation.^[17]^ Also evidence has indicated that the combination therapy of both Clopidogrel and Aspirin reduces the risk of the furthering major ischemic events via up to one-third additionally in STEMI patients undergoing PCI, with no obvious increase in bleeding.^[4]^ However, the role of combination therapy between Aspirin and Clopidogrel in a broader sense with patients being at high risk for cardiovascular events is still unknown. Therefore, we designed this study to best compare the efficacy and prognosis between the single drug therapy (Aspirin) and the combination of the aforementioned drugs on STEMI patients.

## 2 Material and methods

### 2.1 Ethics statement

The study was approved by the Ethical Committee of the Third Hospital of Hebei Medical University. All participants have provided informed written consent.

### 2.2 Study subjects

A random selection of a total of 136 STEMI patients in the Hebei province of China was conducted during the January 2014 to September 2015 time period from the Third Hospital of Hebei Medical University. These patients consisted of 79 males (58.09%) and 57 females (41.91%), with the mean age of 51.70 ± 5.10 years (ranging from 40 to 67 years). The inclusion criteria went as follows according to the results provided by the European Society of Cardiology (ESC) congress 2012 (Munich, Germany): patients diagnosed by a professional pathologist with continuous chest pain >30 minutes and an electrocardiogram (ECG) indicating elevation of 2 or more ST-segments in adjacent leads (chest lead ≥0.2 mV, limb lead ≥0.2 mV) or acute myocardial infarction (AMI) history with left bundle-branch block (affecting ST-segment analysis), onset time <6 hours; in patients with positive cardiac troponin I (cTnI) and creatine kinase isoenzyme MB (CK-MB) increased by 2-fold more than the reference value; patients with precardiogenic shock and severe left main coronary disease; patients with complete medical records and without both radiotherapy and chemotherapy before treatment. The exclusion criteria went as follows: patients who were <21 years of age or >80 years of age; patients with contraindication involving thrombolysis (including previous history of stroke, intracranial tumors, and cerebral hemorrhage), and antiplatelet or antithrombotic drugs; patients with high blood pressure that was not strictly controlled, with cardiogenic shock as well as high risk of bleeding, who had previously undergone coronary artery bypass surgery, and had long-term intake of oral anticoagulant drugs; patients suffering from liver and kidney dysfunction.

### 2.3 Treatment regimens

Patients were randomly assigned into both the observation group and the experimental group. First, after assignment, patients in the 2 groups were given thrombolytic therapy with urokinase (1.5 million U, H12020492; Tianjin Biochem Pharmaceutical Co., Ltd., Tianjin, China), and then treated with lipid-lowering drugs, antihypertensive drugs, nitrates, β-blockers, angiotensin-converting enzyme (ACE) inhibitors, and calcium antagonists in accordance with their specific conditions. Patients in the observation group were treated with 300 mg Aspirin (Sp682; Shanghai H-Y Biological Technology Co., Ltd., Shanghai, China) administrated by chewing once admitted to the hospital and afterward maintained health with 100 mg Aspirin daily. Patients in the experimental group orally took 75 mg Clopidogrel (Boliwei, produced by France Sanofi San Dela Fort Pharmaceutical Co., Ltd., sub-packed by Hangzhou Sanofi St. De La Fort Minsheng Pharmaceutical Co., Ltd.) daily and Aspirin treatment of the same dosage as part of the observation group. Both groups received 4 weeks of treatment for optimal results.

### 2.4 Enzyme-linked immunosorbent assay

Initially, 5 mL of venous blood was collected from each patient in the morning at 6: 30 and centrifuged for 10 minutes. The supernatant was then collected and the levels of interleukin-6 (IL-6), tumor necrosis factor-α (TNF-α), and N-terminal pro-brain natriuretic peptide (NT-proBNP) were all measured in strict accordance with the instructions provided by the ELISA kit (F26231-A, Shanghai Huyu Biological Co., Ltd., Shanghai, China). The high-sensitivity C-reactive protein (hs-CRP) was measured by the rate nephelometry method by making use of a BN II analyzer (Dade Behring, Marburg, Germany) with the original reagent. The operation was carried out in strict accordance with the instructions provided by the kit.^[18]^

### 2.5 Color Doppler ultrasound

After 4 weeks of treatment, a color Doppler ultrasonography was performed using an ultrasound detector (7500, Philips, Inc., China), with a frequency between 2 and 4 MHz to detect the left ventricular end-systolic diameter (LVESD), left ventricular end-diastolic diameter (LVDd), and left ventricular ejection fraction (LVEF).^[19]^

### 2.6 Observation of inflammation, cardiac function, and hemorrhage complication

The levels of inflammatory factors and the change of cardiac function were both observed in comparison before and after 4 weeks of treatment. Venous blood was collected from 2 groups of patients under fasting conditions before and after treatment in the morning. The renal function indicators creatinine (Cr), uric acid (UA), and blood urea nitrogen (BUN) were also detected by a OLYMPUS AU5400 automatic biochemical analyzer, and the levels of cTnI, CK-MB, and creatine kinase (CK) after treatment were also examined. Hemorrhage complications were also observed in the 2 groups during this time. Severe hemorrhage means excessive bleeding and that blood transfusion treatment is imperative or the continuous bleeding may be life-threatening such as retroperitoneal hemorrhage, cerebral hemorrhage, or bleeding leading to decreases in hemoglobin by >50 g/L. Mild hemorrhage is a small amount of bleeding, which can be coagulated by either the general treatment or by itself without any treatment or disable anticoagulant therapy, and moderate hemorrhage is between severe hemorrhage and mild hemorrhage. In case the condition was worse than moderate bleeding, the treatment program will be readjusted.

### 2.7 Follow-up

The follow-up appointments were performed after the therapy until the patients died, lost to follow-up, or the last time of follow-up, which were stopped on March 22, 2016. If the patients were still alive at the end of the follow-up, the censored data were utilized for them. The 9 cases of patients who lost to follow-up were dealt with the last statistic time. The follow-up records were collected through outpatient service, telephone, or referring back to previous medical records. The main records of the follow-up included the occurrence of postinfarction angina, recurrent myocardial infarction, stroke, and death.

### 2.8 Efficacy evaluation

After the treatment, the ECG of the patients in the 2 groups was measured based on their ECG detection results^[20]^ before and after treatment. The criteria for the efficacy evaluation were as follows: marked effect—the sum of ST-segment elevation showed a fall back >70% (>70%) under continuous lead; moderate effect—the sum of ST-segment elevation showed a fall back between 30% and 70% under continuous lead; no effect—the sum of ST-segment elevation showed a fall back <30% (<30%) under continuous lead. The total effect rate = (cases of marked effect + cases of moderate effect)/total number of cases × 100%.

### 2.9 Statistical analysis

A statistical analysis was performed using the SPSS 19.0 software (IBM Corp., Armonk, NY). Measurement data were presented as the mean ± standard deviation. First, Kolmogorov–Smirnov (K–S) test and Levene test were performed to determine normal distribution of data and equality of variances. When the tests revealed normal distribution and equal variance, the comparison inside the group before and after treatment was analyzed using paired *t* test, whereas the comparison among groups was conducted by independent-sample *t* test. When sample variances were unequal or data weren’t normally distributed, the data were analyzed by rank sum test. Enumeration data were expressed as percentage or rate and analyzed using χ^2^ test. Whether the combination of Clopidogrel and Aspirin for STEMI treatment was effective was regarded as a dependent variable, and risk factors related to efficacy were included in the multivariate logistic regression analysis. Two-sided *P* <.05 was considered statistically significant.

## 3 Results

### 3.1 The patients are comparable in the observation and experimental groups

There were 136 patients randomly grouped into the observation (n = 68) and experimental groups (n = 68). Clinical characteristics including age, smoking history, alcohol drinking, hypertension, hyperlipemia, diabetes mellitus, family history, and body mass index (BMI, kg/m^2^) and the administration of β-blockers and ACE inhibitors were all collected from the patients. There were no obvious differences between the 2 groups with regard to these clinical characteristics (*P* >.05) (Table 1), indicating that patients are comparable in the observation and experimental groups.

**Table 1:**
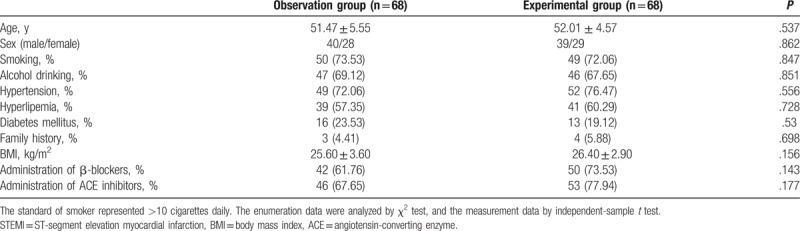
Clinical characteristics of STEMI patients between the observation and experimental groups [n, n (%)].

### 3.2 The patients have alleviated myocardial injury by the combination therapy of Clopidogrel and Aspirin

CK-MB, cTnI, and CK levels can indirectly reflect the degree of myocardial injury. After treatment, we detected the levels of CK-MB, cTnI, and CK in the observation and experimental groups. The results showed that the CK-MB, cTnI and CK peak values in the experimental group were significantly lower than those in the observation group (*P* <.05) (Table 2). Overall, the combination therapy of Clopidogrel and Aspirin could better aid in alleviating myocardial injury of the patients with STEMI.

**Table 2:**
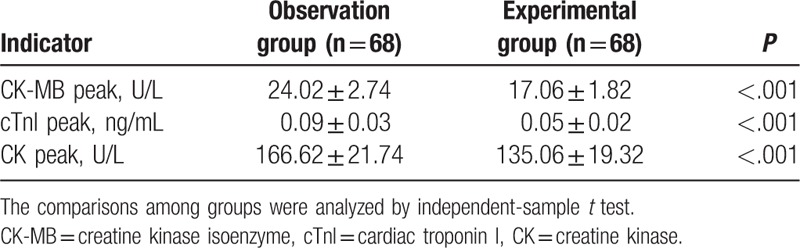
The CK-MB peak, cTnI peak, and CK peak of patients after the different treatments in the observation and experimental groups.

### 3.3 The patients have better clinical efficacy by the combination therapy of Clopidogrel and Aspirin

After treatment, we detected the clinical efficacy of the patients in the observation and experimental groups, the outcome of which revealed that patients in the observation group had 19 cases with marked effect, 30 cases with moderate effect, and 19 cases with less or no effect, with the overall response rate being 72.06%. In the experimental group, there were 37 cases of marked effect, 28 cases of moderate effect, and 3 cases with little to no effect, with the overall response rate being a near guarantee of 95.59%. These results indicated that the patients had better clinical efficacy in the experimental group than in the observation group after treatment (*P* <.05) (Table 3). Therefore, the combination therapy of Clopidogrel and Aspirin might have the potential to contribute to a better clinical therapeutic effect.

**Table 3:**
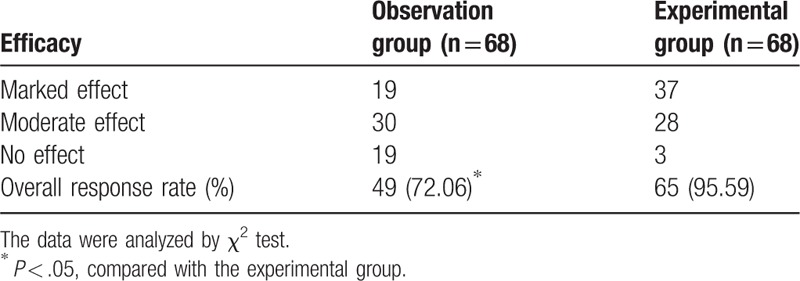
The patients have better clinical efficacy after the combination therapy of Clopidogrel and Aspirin.

### 3.4 The patients have improved cardiac function by the combination therapy of Clopidogrel and Aspirin

Before treatment, no obvious differences were observed in the levels of LVESD, LVEF, and LVDd between the experimental and observation groups. After 4 weeks of treatment, LVESD and LVDd were reduced in the observation group and the experimental group, and LVEF was elevated; significant differences were detected in regard to all these indicators between the 2 groups after treatment (all *P* <.05) (Table 4), making the indication that the cardiac functions of patients in the 2 groups were improved after treatment and that the experimental group showed a better efficacy after the combination therapy of Clopidogrel and Aspirin.

**Table 4:**
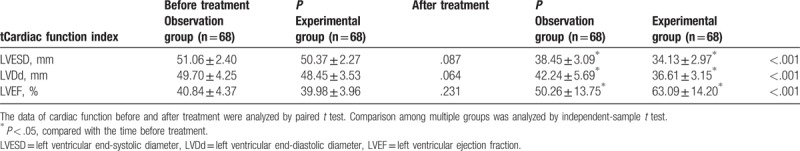
The patients have improved cardiac function after the combination therapy of Clopidogrel and Aspirin.

### 3.5 The patients have improved renal function after the combination therapy of Clopidogrel and Aspirin

Before treatment, no obvious differences were observed in the levels of Cr, UA, and BUN between the experimental and observation groups. After 4 weeks of treatment, however, the renal function in both groups was better than that before treatment, and the experimental group exhibited better renal function in comparison with the observation group (all *P* <.05) (Table 5), making the indication that the combination therapy of Clopidogrel and Aspirin could better improve renal function of patients suffering from STEMI.

**Table 5:**
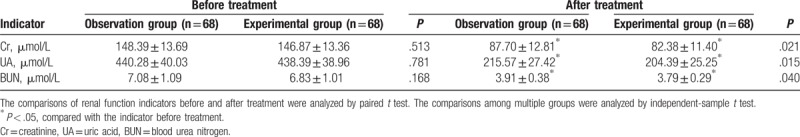
The patients have improved renal function after the combination therapy of Clopidogrel and Aspirin (mean ± standard deviation).

### 3.6 The patients have lower levels of IL-6, TNF-α, NT-proBNP, and hs-CRP by the combination therapy of Clopidogrel and Aspirin

As shown in Table 6, there were no obvious differences found in serum levels of inflammatory factors (IL-6, TNF-α, NT-proBNP, and hs-CRP) between the experimental and observation groups before treatment. The serum levels of inflammatory factors (IL-6, TNF-α, NT-proBNP, and hs-CRP) in the 2 groups showed a decline after treatment. The serum levels of IL-6, TNF-α, NT-proBNP, and hs-CRP in the observation group were also significantly higher than those in the experimental group (*P* <.05). Therefore, the combination therapy of Clopidogrel and Aspirin had a better effect on the inhibition of inflammation.

**Table 6:**
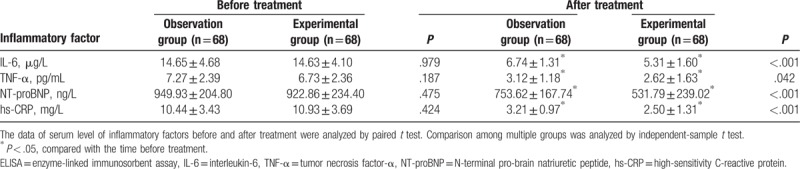
Serum levels of inflammatory factors detected by ELISA between the observation and experimental groups before and after treatment.

### 3.7 LVESD, LVDd, and the levels of IL-6, TNF-α, NT-proBNP, and hs-CRP are risk factors for STEMI

The efficacy of the combination of Clopidogrel and Aspirin for STEMI treatment was evaluated as a dependent variable, and the IL-6, TNF-α, NT-proBNP, hs-CRP, LVESD, LVDd, and LVEF, Cr, UA, BUN were all included in the multivariate logistic regression analysis. The results indicated that LVEF was a favorable factor for support of the combination therapy of Clopidogrel and Aspirin in patients with STEMI (*P* <.05), whereas LVESD, LVDd, IL-6, TNF-α, NT-proBNP, and hs-CRP presented to be risk factors (*P* <.05). Cr, UA, and BUN were detected to have no significant effect on the combination therapy of Clopidogrel and Aspirin in patients with STEMI (*P* >.05) (Table 7).

**Table 7:**
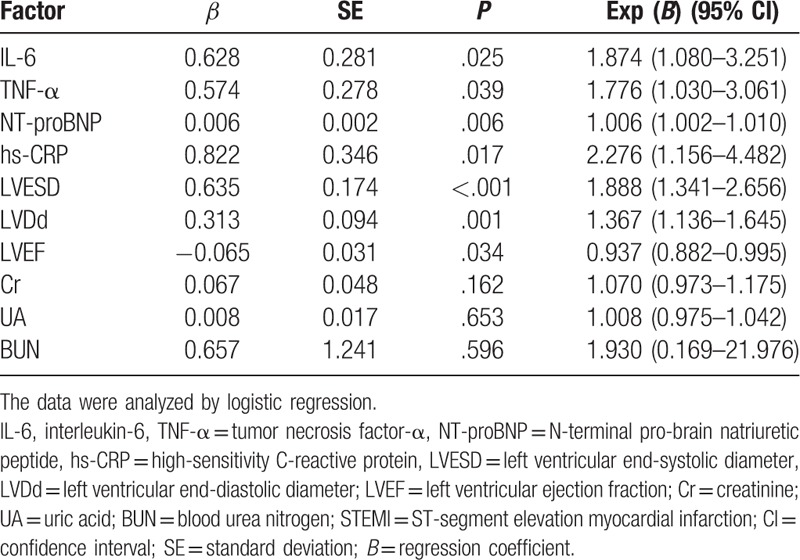
Logistic regression analysis to identify risk factors for the combination therapy of Clopidogrel and Aspirin for STEMI.

### 3.8 The incidence of hemorrhagic complication does not differ between the observation and experimental groups after treatment

The incidence of hemorrhagic complication in the observation and experimental groups after treatment was subsequently detected. There were no cases observed with a severe hemorrhage in the 2 groups after treatment. There were, however, 3 cases of mild hemorrhage and 3 cases of moderate hemorrhage in the observation group, whereas there were 4 cases of mild hemorrhage and 3 cases of moderate hemorrhage in the experimental group. The hemorrhagic symptoms disappeared after general treatment in both groups, and there were no marked differences in the hemorrhagic complication between the 2 groups (*P* >.05), as shown in Table 8, reflecting the feasibility of combination therapy of Clopidogrel and Aspirin for the treatment of STEMI.

**Table 8:**
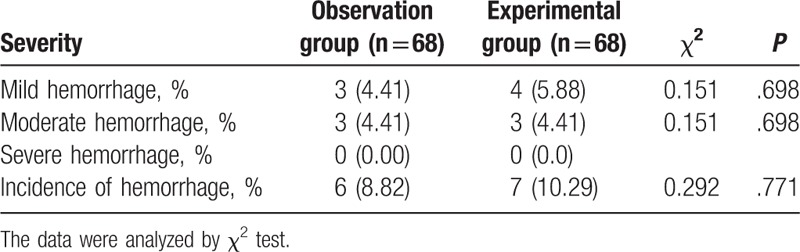
The comparison of hemorrhagic complication in the observation and experimental groups after treatment [n, n (%)].

### 3.9 The patients have lower incidence of cardiac events by the combination therapy of Clopidogrel and Aspirin

In addition, the incidence of cardiac events in the observation and experimental groups after treatment was detected: the incidence of post-infarction angina (2.94%), recurrent myocardial infarction (1.47%), stroke (0), and death (1.47%) in the experimental group were all obviously lower than those occurring in the observation group according to the follow-up results (Table 9), suggesting a relatively better prognosis of the combination therapy for STEMI.

**Table 9:**
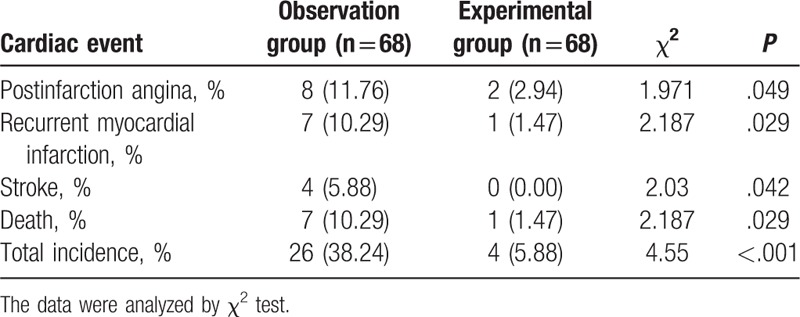
The comparison of incidence of cardiac events in the observation and experimental groups after treatment [n, n (%)].

## 4 Discussion

Cardiovascular disorders such as MI are the leading causes of mortality around the world.^[21]^ MI is linked to an inflammatory reaction occurring during the healing and scar formation process.^[22]^ Antiplatelet drugs play a preventive role in the progression of patients with MI, particularly in association with PCI, including STEMI.^[23]^ Therefore, this study placed focus on 2 kinds of antiplatelet drugs, Clopidogrel and Aspirin with the aim being to investigate their role in management of patients with STEMI in the Hebei province of China, and discover the combination therapy of Clopidogrel and Aspirin being beneficial in patients with STEMI.

Initially, we found that the CK-MB, cTnI, and CK peak values in the experimental group were significantly lower than those in the observation group after treatment, indicating that the combination therapy of Clopidogrel and Aspirin could better aid in alleviating myocardial injury of the patients with STEMI. CTnI and CK-MB are considered plasma level markers for myocardial injury.^[24]^ It has been reported that Clopidogrel pretreatment can provide a notably reduced incidence of troponin elevation following elective PCI.^[25]^ In addition, we detected that combination therapy of Clopidogrel and Aspirin reduced the levels of LVESD, LVDd, and elevated LVEF indicating the promotion of the cardiac function of STEMI patients. Mean platelet volume (MPV) is an indicator of both platelet activation and platelet functions.^[26]^ LVESD, LVDd, and LVEF refer to indicators of left ventricular remodeling, and the development of left ventricular remodeling after MI is a predictor of heart failure and mortality.^[27]^ As Clopidogrel and Aspirin are the main antiplatelet agents involved in this study, there may exist a negative correlation with the MPV and a positive correlation with left ventricular remodeling. Acar et al have demonstrated an indirect role MPV plays in left ventricular remodeling, for this reason, a high MPV may play an alerting role for the possible left ventricular dysfunction in acute anterior STEMI patients.^[28]^ Besides, coherent with our study, a study proposed by Liu et al has revealed that Clopidogrel in addition to Aspirin decreases the major cardiac and cerebrovascular events involved in patients with STEMI, which may be protective against cardiac function.^[29]^ Furthermore, after treatment, we found that the levels of Cr, UA, and BUN were better improved in the experimental group. Cr, UA, and BUN can be used to evaluate renal functions.^[30]^ As previously reported, unlike the use of only saline-endotoxin, the use of Clopidogrel-endotoxin did not result in the deterioration of Cr clearance.^[31]^ Moreover, the addition of Clopidogrel to standard treatment in non-ST elevation acute coronary syndrome was found to be beneficial for renal function.^[32]^

In addition, the combination therapy of Clopidogrel and Aspirin decreased serum levels of IL-6, TNF-α, NT-proBNP, and hs-CRP in STEMI patients. A study conducted by Hwang et al has revealed that the increased level of IL-6 may play a pivotal role in the development of stent thrombosis (ST), even in patients treated with potent antiplatelet agents such as Clopidogrel.^[33]^ In human glioblastoma, the decreased level of IL-6 induced by Aspirin may contribute to cell apoptosis.^[34]^ A study conducted by Antonino et al concluded that the long-term Clopidogrel therapy reduced platelet activity is also associated with an anti-inflammatory effect leading to decreases in level of IL-6, IL-2, TNF-α, and TNF-β.^[35]^ Based on conventional anti-ischemic cardiomyopathy therapy, Trimetazidine combined with Clopidogrel improved heart function effectively while also decreasing NT-proBNPa levels of ischemic cardiomyopathy (ICM) patients.^[36]^ Hs-CRP is an inflammatory factor and the median level of hs-CRP in serum was significantly suppressed after therapy with Clopidogrel in patients undergoing PCI.^[37,38]^ Aspirin has proven to diminish the levels of IL-6 and hs-CRP in acute coronary syndrome (ACS) patients.^[39]^ In addition, Aspirin can suppress TNF-alpha-stimulated fractalkine expression in a dose-dependent manner through the nuclear factor-kappa B p65 pathway in human umbilical vein endothelial cells.^[40]^ Based on these findings, we speculated that the combination therapy of Clopidogrel and Aspirin may suppress inflammation in STEMI with reduced levels of major inflammatory factors.

Consequently, we demonstrated that Clopidogrel combined with Aspirin reduced the incidence of posttreatment cardiac events including postinfarction angina, recurrent myocardial infarction, stroke, and death. The study conducted by Sabatine et al has declared that Clopidogrel and Aspirin combined therapy leads to a significant reduction in the odds of death from cardiovascular causes, recurrent myocardial infarction, and recurrent ischemia of STEMI patients.^[41]^ It has proven that the combination therapy of Clopidogrel and Aspirin is widely used in patients with ACS and undergoing PCI.^[42]^ The reduction in the rate of ischemic events by means of antiplatelet agents, including both oral agents (Aspirin and Clopidogrel), has uniformly been confirmed to accompany with an increase in bleeding.^[43]^ Both Clopidogrel and Aspirin have a potent protective effect against adverse vascular events, what's more, the combination of these 2 agents has an even stronger antiplatelet effect translating into superior antithrombotic protection in coronary, cerebral, or peripheral arterial disease, without even an inordinate increase in bleeding complications.^[44]^ Sabatine et al also found that in patients 75 years of age or younger diagnosed with STEMI and exclusively receive Aspirin as the primary treatment option, the addition of Clopidogrel will improve the patency rate of the infarct-related artery and reduce ischemic complications.^[41]^ More importantly, the combination of Clopidogrel and Aspirin has a satisfactory curative efficacy for acute STEMI patients.^[43,45]^ Consistent with these findings, our study also verified no significant increase in bleeding by the combination therapy of Clopidogrel and Aspirin than a single Aspirin treatment, with a better prognosis in patients with STEMI.

In conclusion, our study manifested that the combination therapy of Clopidogrel and Aspirin provided an improvement on the cardiac function and prognosis of STEMI patients in the Hebei province of China, providing less inflammation as well. This goes to show the importance of a combination therapy of Clopidogrel and Aspirin and its significant contribution toward treatment of STEMI, which offers better treatment outcome than single use of Aspirin. However, a small sample size together with a limited region and population lead to an uncertainty in the final investigation results. There are still some important issues that remain to be further investigated. This clinical study may be furthered with investigation at molecular level, and relevant therapeutic targets and signaling pathways may be identified in future studies. Moreover, the occurrence of drug-resistance in some patients for the antithrombotic agents (Clopidogrel and Aspirin) can and still is a possible challenge. Still, the evidence provided by the study supports the stance that the combination therapy of Clopidogrel and Aspirin is likely to present as a potential effective strategy in the treatment of STEMI.

## Acknowledgments

We would like to give our sincere appreciation to the reviewers for their helpful comments on this article.

## Author contributions

**Conceptualization:** Hai-Rong Yu, Yue-Yue Wei, Jian-Guo Ma, Xiao-Yong Geng.

**Data curation:** Hai-Rong Yu, Yue-Yue Wei, Jian-Guo Ma, Xiao-Yong Geng.

**Formal analysis:** Hai-Rong Yu, Jian-Guo Ma, Xiao-Yong Geng.

**Investigation:** Hai-Rong Yu, Yue-Yue Wei, Jian-Guo Ma, Xiao-Yong Geng.

**Methodology:** Hai-Rong Yu, Yue-Yue Wei, Jian-Guo Ma.

**Project administration:** Hai-Rong Yu, Yue-Yue Wei, Xiao-Yong Geng.

**Resources:** Hai-Rong Yu.

**Software:** Yue-Yue Wei, Xiao-Yong Geng.

**Supervision:** Hai-Rong Yu, Yue-Yue Wei, Jian-Guo Ma, Xiao-Yong Geng.

**Validation:** Hai-Rong Yu, Yue-Yue Wei, Jian-Guo Ma.

**Visualization:** Hai-Rong Yu, Yue-Yue Wei, Jian-Guo Ma, Xiao-Yong Geng.

**Writing – original draft:** Hai-Rong Yu, Yue-Yue Wei, Jian-Guo Ma.

**Writing – review and editing:** Hai-Rong Yu, Yue-Yue Wei, Jian-Guo Ma, Xiao-Yong Geng.
